# The monocyte counts to HDL cholesterol ratio in obese and lean patients with polycystic ovary syndrome

**DOI:** 10.1186/s12958-018-0351-0

**Published:** 2018-04-10

**Authors:** Akin Usta, Eyup Avci, Cagla Bahar Bulbul, Hasan Kadi, Ertan Adali

**Affiliations:** 1 Department of Obstetrics and Gynecology, School of Medicine, Balıkesir University Faculty of Medicine, Cagis Yerleskesi, Bigadic yolu 17. km, 10145 Balikesir, Turkey; 2 Department of Cardiology, School of Medicine, Balikesir University, Balikesir, Turkey

**Keywords:** PCOS, Monocyte count to HDL cholesterol ratio, Inflammation, Insulin resistance, Cardiovascular disease

## Abstract

**Background:**

Women with polycystic ovary syndrome are more likely to suffer from obesity, insulin resistance, and chronic low-grade inflammation. In fact, the excessive activation of monocytes exacerbates oxidative stress and inflammation. However, high-density lipoprotein cholesterol neutralizes the pro-inflammatory and pro-oxidant effects of monocytes. The aim of this study is to investigate whether monocyte counts to high-density lipoprotein cholesterol ratio can predict the inflammatory condition in patients with polycystic ovary syndrome.

**Methods:**

In this cross-sectional study, a total of 124 women (61 of them with polycystic ovary syndrome and 63 age-matched healthy volunteers) were included in the study population. Obese polycystic ovary syndrome patients (*n* = 30) with a body mass index of ≥25 kg/m^2^ and lean polycystic ovary syndrome patients (*n* = 31) with a body mass index of < 25 kg/m^2^ were compared to age-and body mass index-matched healthy subjects (30 obese and 33 non-obese).

**Results:**

The monocyte counts to high density lipoprotein cholesterol values in women with polycystic ovary syndrome were significantly higher than in control subjects (*p* = 0.0018). Moreover, a regression analysis revealed that body mass index, the homeostasis model assessment of insulin resistance and the high sensitivity C-reactive protein levels were confounding factors that affected the monocyte counts to high density lipoprotein cholesterol values. Additionally, a univariate and multivariate logistic regression analysis demonstrated that the increased monocyte counts to high density lipoprotein cholesterol values were more sensitive than the other known risk factors (such as increased body mass index, homeostasis model assessment of insulin resistance and high sensitive C-reactive protein levels) in the prediction of the inflammation in patients with polycystic ovary syndrome.

**Conclusion:**

The present study demonstrated that the monocyte count to high density lipoprotein cholesterol may be a novel and useful predictor of the presence of polycystic ovary syndrome.

## Background

Polycystic ovary syndrome (PCOS) is a common endocrine disorder that affects 6-20% of women of reproductive age [1, 2]. The disorder is characterized by oligo/anovulation, hormonal and/or clinical hyperandrogenism, and the appearance of polycystic ovaries on ultrasound [1, 2].

Women with PCOS are more likely to suffer from obesity, insulin resistance, glucose intolerance, endothelial dysfunction, hyperandrogenism, and chronic low-grade inflammation than are women without the condition. Chronic low-grade inflammation seems to have a stimulatory effect on the development and progression of endothelial dysfunction and atherosclerosis [3], and long-term complications include type 2 diabetes mellitus and cardiovascular disease [1, 2]. It is therefore important to understand the underlying molecular and cellular mechanisms of this syndrome.

The high-sensitivity C-reactive protein (hsCRP) has been found to be a highly sensitive inflammatory biomarker that is commonly used in the detection of subclinical inflammation in patients with PCOS [4]. The hsCRP test is also used to detect future cardiovascular disease risk [5]. Moreover, increased counts of leucocytes or related subgroups have been found to be additional independent markers as well as prognostic factors in the development of inflammation and atherosclerosis [6, 7]. Regarding the initiation and progression of the atherosclerotic process, several biochemical markers (to include hsCRP and leukocyte counts) associated with inflammatory conditions, oxidative stress, and endothelial dysfunction have been shown to reflect the severity and prognosis of cardiovascular disease risk.

Monocytes constitute roughly 3-8% of circulating leukocytes and-in conjunction with other granular and agranular cells such as eosinophils, basophils, neutrophils, and lymphocytes-are essential components of the innate immune system. In tissues, circulating monocytes and their differentiated forms, known as macrophages, play central roles in the initiation and resolution of inflammation; this is principally achieved via phagocytosis, the release of inflammatory cytokines, the presence of reactive oxygen species, and the activation of the acquired immune system [8]. However, the accumulation of monocytes exacerbates oxidative stress and inflammation. Thus the excessive activation of monocytes aggravates diseases such as atherosclerosis, arthritis, and multiple sclerosis, primarily affecting platelets and endothelial cells and leading to the activation of the prothrombotic pathway [9].

In contrast, high-density lipoprotein cholesterol (HDL-C) neutralizes the pro-inflammatory and pro-oxidant effects of monocytes by hindering the oxidation of low-density lipoprotein (LDL) molecules and the migration of macrophages as well as by promoting the efflux of cholesterol from these cells [10]. In addition to outlining the well-known anti-inflammatory and anti-oxidant actions of HDL-C particles, it has recently been claimed that these molecules play a suppressive role in the control of monocyte activation and in the proliferation and differentiation of the progenitor cells of monocytes [11].

Recognised as a recently-emerged inflammation-based marker, the monocyte count to HDL-C ratio (MHR) has been reported to be a new predictor and prognostic indicator of mortality and morbidity in many diseases [12, 13]. The MHR has also been found to be correlated with hsCRP levels in diseases that are associated with chronic inflammation, such as cardiovascular disease [14], chronic kidney disease [12], abdominal aortic aneurysm [15], intracerebral hemorrhage [16], hypertension [17], and metabolic syndromes [18].

According to the Rotterdam criteria [1], the prevalence of PCOS in Turkish women is approximately 20% [2]. Thus, a considerable number of Turkish women with PCOS are at an increased risk of developing diabetes and cardiovascular disease in the future. The early diagnosis and prevention of inflammatory conditions in the PCOS population is essential to maintain the long-term wellbeing of these women and will aid in the proper utilization of public health resources. To the best of our knowledge, no study has yet examined the association between PCOS and the MHR. Therefore, we aimed to investigate whether the MHR, a readily available inflammatory and oxidative stress marker, is associated with PCOS.

## Methods

A cross-sectional study was conducted at the Balikesir University, School of Medicine, Department of Obstetrics and Gynecology and Department of Cardiology between January 2017 and June 2017. The study was approved by the Ethics Committee of Balikesir University, and study protocols of were in accordance with the Helsinki Committee requirements. Informed consent was obtained from all participants.

In total, 124 women (61 with PCOS and 63 age-matched healthy volunteers) were included in the study population. Obese PCOS patients (*n* = 30) with a body mass index (BMI) of ≥25 kg/m^2^ and lean PCOS patients (*n* = 31) with a BMI of < 25 kg/m^2^ were compared to healthy age-and BMI-matched subjects (30 obese and 33 non-obese). All patients were aged between 18 and 40 years.

PCOS was diagnosed using the Rotterdam criteria [1]. According to these diagnostic criteria, the presence of oligomenorrhea (wherein menstrual cycles occur more than 35 days apart) or amenorrhea (wherein menstrual cycles ocur at least six months apart) was defined as oligoovulation, and the presence of at least 12 follicles measuring 2–9 mm in diameter and/or an ovarian volume greater than 10 cm^3^ in per ovary indicated the existence of polycystic ovaries; additionally, the presence of hirsutism was evaluated using the Ferriman–Gallwey scoring system [19]. Areas near the lip, chin, chest, upper and lower abdomen, upper arm, forearm and thigh were visually assessed and granded a score between zero (the absence of terminal hair) and four (the presence of male-pattern terminal hair). A score greater than or equal to six was defined as hirsutism.

Patients who were diagnosed with other disorders whose clinical features are similar to those of PCOS, to include Cushing’s syndrome, congenital adrenal hyperplasia and androgen-secreting tumors, were excluded from participation. Additionally, patients who took medicines, such as oral contraceptives, antilipidemic and/or antihypertensive medications, steroids, anti-diabetic medications, anticoagulants, or antiplatelet drugs were excluded from the study.

Each participant’s height (m) and weight (kg) were measured while the patient was dressed only in underwear clothing. Waist circumference was measured as the minimum distance between the iliac crest and the lateral costal margin. The waist-to-hip ratio (WHR) was calculated as the ratio of the waist measurement to that of the hip. BMI was calculated as the patient’s mass in kilograms divided by the square of the patient’s body height in meters (kg/m^2^). All participant data was recorded and stored in a computer database.

All PCOS and control patients were pooled from university’s gynecology clinic. Additionally, PCOS patients were reevaluated to determine whether they truly met the clinical, ultrasound scan (U/S), and biochemical criteria for PCOS based on the Rotterdam criteria. Physical and gynecological examinations, pelvic ultrasounds, and peripheral venous blood samplings were performed during Day 2 or 3 of a participant’s menstrual cycles or progestogen induced early follicular phase (Day 2 or 3) in subjects with amenorrhea. All women were examined, and pelvic ultrasound scans were performed using a 7.0 MHz vaginal transducer (Voluson 730, GE Healthcare, USA) by the same gynecologist.

During blood pressure measurements, the brachial artery was occluded by a cuff placed around the patient’s upper arm; the cuff was then inflated to above systolic pressure. As the cuff gradually deflated, pulsatile blood flow was reestablished and was accompanied by sounds that were able to be detected by a stethoscope held over the artery just below the cuff. The onset of the pulsatile blood flow sound (Korotkoff I) was recorded as systolic blood pressure and the disappearance of blood flow sounds (Korotkoff V) was recorded as diastolic blood pressure. All measurements were performed by healthcare professionals, who were blinded to the clinical diagnoses of the patients.

### Biochemical evaluation

After overnight fasting, blood samples were collected from the antecubital vein of patients between the hours of 9 and 10 AM. Serum was collected following centrifugation at 2500 g for 10 min, and stored in deep freeze at − 80 °C to await biochemical and hormonal assessment. To avoid possible assay variability, all patient blood samples were analyzed together.

Complete blood counts were measured via Sysmex XE-2100 (Sysmex Corp. Kobe, Japan) using either the fluorescence flow cytometry or electrical impedance method. The serum levels of follicle-stimulating hormone (FSH), luteinizing hormone (LH), estradiol (E2), thyroid-stimulating hormone (TSH), and total testosterone were determined using commercially avaliable enzyme-linked immunosorbent assay (ELISA) kits (eBioscience, Austria) on a diagnostic instrument (BioTek, ELx 800, USA). 2nd International reference preparation was used for FSH and LH measurements. The levels of glucose, total cholesterol, low-density lipoprotein (LDL) cholesterol, and high-density lipoprotein (HDL) cholesterol were measured using commercially available kits on a chemistry AutoAnalyzer (Cobas Integra 800; Roche Diagnostics GmbH; Mannheim, Germany). Serum high sensitive C-reactive protein (hs CRP) was measured with chemiluminescent immunoassay using an ADVIA Centaur XP (Siemens Healthcare Diagnostics, NY, USA). The levels of fasting insulin were determined using commercial kits and an automatic hormone analyzer (Beckman Coulter; Unicel DXI 600; Access Immunoassay System). The homeostasis model assessment for insulin resistance (HOMA-IR) was defined as (insulin x glucose) / 22.5 as previously described [6].

### Statistical analysis

The MedCalc Statistical Software Program version 17.2 (MedCalc, Belgium) was used for statistical analysis. Normally distributed data are described as mean ± standard deviation, otherwise, as median (minimum-maximum). Whether the distributions of continuous variables were normal or not was determined by Kolmogorov–Smirnov test. Also, the Levene test or F test was used for the evaluation of homogeneity of variances.

The student’s t test was used to compare normally distributed measurements for independent samples and the Mann–Whitney U test was applied for comparisons of the median values. The Chi-square test was used to compare categorical data. While the mean differences among more than two independent groups were analyzed by one-way ANOVA, the Kruskal–Wallis test was applied for comparisons of the median values. When the *p* value from one-way ANOVA or Kruskal–Wallis test statistics was statistically significant the Scheffé test or Post-Hoc analysis nonparametric multiple comparison test was used to determine which group differed from which others. The possible confounding factors that associated with MHR value were evaluated by using logistic regression analysis. In order to determine of the independent variables for presence of PCOS, the univariate and multivariate logistic regression analysis were performed. To evaluate model characteristics of fitness, Hosmer–Lemeshow tests were used. A *p*-value of < 0.05 was considered statistically significant.

## Results

A total of 124 women—61 with PCOS (30 obese, 31 non-obese) and 63 age- and BMI matched controls (30 obese, 33 non-obese)—were included in this study. The mean age of participants was 23.9 ± 4.9 in the PCOS group and 25.2 ± 5.1 in the control group; there was no statistically significant difference (*p* = 0.1566) between the groups. The mean BMI of participants was 26.6 ± 6.1 in the PCOS group, and 24.8 ± 5.3 in the control group (*p* = 0.0858).

As compared to the control subjects, PCOS patients had significantly higher WHRs, Ferriman Gallwey scores, diastolic blood pressure levels, insuline levels, HOMA-IR readings, hsCRP scores, total leucocytes counts, neutrophil counts, monocyte counts, total cholesterol levels, LDL levels, total testosterone levels, LH levels and MHRs (*p* < 0.05). However, HDL-C levels were lower in PCOS patients than in control subjects (*p* < 0.0001). The clinical, hormonal, and biochemical characteristics of the groups are summarized in Table 1.

**Table 1:** The clinical, hormonal, and biochemical characteristics of patients in tje PCOS and non-PCOS groups

	PCOS	Non-PCOS	*P* value
Age (year)	23.9 ± 4.9	25.2 ± 5.1	0.1566
Body mass index (kg/m^2^)	26.6 ± 6.1	24.8 ± 5.3	0.0858
Waist to hip ratio (WHR) (cm)	0.80 ± 0.07	0.77 ± 0.06	0.0030
Ferriman–Gallwey score	5 (2-17)	3 (1-6)	< 0.0001
Systolic blood pressure (mmHg)	120 (90-150)	110 (90-130)	0.1195
Diastolic blood pressure (mmHg)	70 (60-110)	70 (60-80)	0.0115
Glucose (mg/dl)	88 (64-123)	81 (61-110)	0.0092
HOMA-IR	2.42 (0.6-13.8)	1.49(0.4-8.8)	0.0027
hsCRP (mg/l)	3.2 (0.3-29.0)	1.8 (0.2-7.8)	0.0074
Leucocytes (/mm^3^)	7.86 ± 2.28	6.92 ± 2.19	0.0208
Neutrophil (/mm^3^)	4.76 ± 1.68	4.08 ± 1.35	0.0150
Lymphocytes (/mm^3^)	2.48 ± 0.76	2.29 ± 0.65	0.0820
Monocyte (/mm^3^)	645.9 ± 205.8	510.9 ± 184.9	0.0002
Trigliserid (mg/dL)	95.0 (46.0-529.0)	78.0 (44.0-280.0)	0.1080
Total cholesterol (mg/dL)	180.1 ± 33.6	168.1 ± 28.5	0.0221
LDL (mg/dL)	102.1 ± 29.7	91.2 ± 25.6	0.0373
HDL (mg/dL)	47.5 ± 10.8	58.7 ± 13.4	< 0.0001
Total testosteron (ng/dl)	1.20 (0.10-4.71)	0.15 (0.07-0.90)	< 0.0001
FSH (IU/L)	5.86 ± 1.37	6.34 ± 1.68	0.0818
LH (IU/L)	9.15 (5.03-22.10)	6.40 (4.72-14.60)	< 0.0001
Estradiol (pg/ml)	45.32 ± 13.67	41.87 ± 14.21	0.1705
TSH (mIU/L)	2.15 ± 1.17	2.08 ± 1.03	0.7119
Prolaktin (ng/ml)	17.34 ± 8.17	16.27 ± 5.89	0.4072
Monocytes to HDL ratio	11.46 (3.70-30.50)	8.77 (3.31-24.19)	0.0018

Data was showed: mean ± standard deviation (SD) or median (min-max)

The biochemical parameters of obese and lean patients with or without PCOS are shown in Table 2. Obese PCOS patients had higher levels of diastolic blood pressure, HOMA-IR, hsCRP, monocytes, total cholesterol, and LDL-C but lower levels of HDL-C than did control participants (*p* <  0.05). Lean PCOS patients had higher levels of HOMA-IR, hsCRP, neutrophils, monocytes, and total testosterone but lower levels of HDL-C and lymphocytes than did control participants (*p* <  0.05).

**Table 2:** The clinical and laboratory parameters of obese and lean patients with or without PCOS

	Obese PCOS *n* = 30	Lean PCOS *n* = 31	Obese Control *n* = 30	Lean Control *n* = 33	*p* value
Age (year)	24.6 ± 4.6	24.7 ± 4.9	23.4 ± 4.3	26.9 ± 5.3	0.059
Body mass index (kg/m^2^)	29.1 ± 4.2 ^a,c^	22.3 ± 3.7 ^d^	31.1 ± 4.6 ^f^	21.4 ± 2.8	< 0.001
Waist to hip ratio (WHR) (cm)	0.85 ± 0.06 ^a,b,c^	0.75 ± 0.04 ^d,e^	0.80 ± 0.06 ^f^	0.73 ± 0.03	< 0.001
Ferriman–Gallwey score	7 (3-17) ^a,b,c^	4 (2-13) ^d,e^	2 (1-5)	1 (1-4)	< 0.001
Systolic blood pressure (mmHg)	120 (90-150) ^a,c^	110 (90-130)	110 (100-130)	110 (90-120)	0.011
Diastolic blood pressure (mmHg)	80 (60-110) ^a,b,c^	70 (60-80)	70 (60-80)	70 (60-80)	0.006
Glucose (mg/dl)	92 (74-123) ^a,b,c^	83 (64-112)	85 (65-110)	78 (61-98)	0.007
HOMA-IR	3.5 (1.4-13.8) ^a,b,c^	2.2 (0.6-9.8)^d,e^	2.6 (1.1-8.8) ^f^	1.2 (0.4-5.6)	< 0.001
hsCRP (mg/l)	4.1(1.2-29.1) ^a,b,c^	1.5 (0.3-8.8) ^e^	2.7 (0.9-7.8) ^f^	0.9 (0.2-3.5)	< 0.001
Leucocytes (/mm^3^)	8.9 ± 2.3 ^a,c^	6.9 ± 1.8 ^d^	7.8 ± 2.6	6.2 ± 1.4	< 0.001
Neutrophil (/mm^3^)	5.1 ± 1.9 ^c^	4.5 ± 1.3 ^e^	4.5 ± 1.4	3.7 ± 1.2	0.005
Lymphocytes (/mm^3^)	2.3 ± 0.7	2.1 ± 0.6	2.5 ± 0.8	2.5 ± 0.7	0.158
Monocyte (/mm^3^)	657.7 ± 259.3 ^b,c^	634.6 ± 139.1 ^d,e^	517.4 ± 184.9	504.7 ± 187.9	0.005
Trigliserid (mg/dL)	145.5(46.0-529.0) ^a,c^	69.0 (53.0-136.0) ^d^	116.0 (58.0-280.0) ^f^	64.0 (44.0-170.0)	< 0.001
Total cholesterol (mg/dL)	189.6 ± 32.1 ^a,b^	164.1 ± 27.5	167.1 ± 26.2	172.2 ± 30.3	0.005
LDL (mg/dL)	110.7 ± 30.5 ^b,c^	95.7 ± 34.6	94.9 ± 22.7	87.8 ± 26.5	0.015
HDL (mg/dL)	43.6 ± 8.9 ^a,b,c^	51.2 ± 11.3 ^e^	54.5 ± 9.9 ^f^	62.8 ± 14.6	< 0.001
Total testosteron (ng/dl)	1.40 (0.32-4.71) ^a,b,c^	1.04 (0.12-2.80) ^d,e^	0.58 (0.07-0.98) ^f^	0.11 (0.07-0.25)	< 0.001
FSH (IU/L)	5.75 ± 1.24	5.97 ± 1.49	6.22 ± 1.60	6.46 ± 1.77	0.302
LH (IU/L)	9.80 (5.03-22.1) ^b,c^	9.15 (5.50-14.5) ^d,e^	6.88 (4.76-14.6) ^f^	5.70 (4.72-10.01)	< 0.001
Estradiol (pg/ml)	47.37 ± 22.64	43.35 ± 18.44	43.26 ± 21.03	41.60 ± 19.47	0.297
TSH (mIU/L)	2.03 ± 1.27	2.27 ± 1.07	1.92 ± 0.91	2.22 ± 1.13	0.564
Prolaktin (ng/ml)	16.42 ± 7.68	18.22 ± 8.42	15.65 ± 6.72	17.15 ± 6.10	0.442
Monocytes to HDL ratio	15.6 (3.7-30.5) ^a,b,c^	9.6 (5.4-24.7) ^e^	9.9 (3.8-20.9) ^f^	7.9 (3.3-24.2)	< 0.001

Data was present: mean ± standard deviation (SD) or median (min-max)

^a^ Obese PCOS vs. Nonobese PCOS (*p* <  0.05)

^b^ Obese PCOS vs. Obese Control (*p* <  0.05)

^c^ Obese PCOS vs. Nonobese Control (*p* <  0.05)

^d^ Nonobese PCOS vs. Obese Control (*p* <  0.05)

^e^ Nonobese PCOS vs Nonobese Control (*p* < 0.05)

^f^ Obese Control vs. Nonobese Control (*p* < 0.05)

Interestingly, we found that obese PCOS patients had higher levels of systolic blood pressure, diastolic blood pressure, HOMA-IR, hsCRP, and total leucocytes but lower HDL-C levels than did lean PCOS patients (*p* <  0.05).

Importantly, we found that MHR values were significantly higher in patients with PCOS than in control subjects (*p* = 0.0018). Moreover, subgroup analysis showed that MHR values of obese PCOS patients were significantly higher than those of lean PCOS patients. Also, MHR values of obese control patients were significantly higher than lean control subjects.

A univariate regression analysis showed that BMI, diastolic blood pressure, HOMA-IR, and hsCRP, were possible confounding factors affecting MHR values (Table 3). Furthermore, in order to identify independent variables that might predict the presence of PCOS, univariate and multivariate logistic regression analyses were performed (Table 4). During the univariate logistic regression analysis, HOMA-IR (odds ratio [OR]: 1.3133 [95% CI: 1.0543–1.6358]; *p* = 0.0067), hsCRP (OR:1.2996 [95% CI: 1.0804–1.5634]; *p* = 0.0023) and the MHR (OR: 1.1006 [95% CI: 1.0314–1.1745]; *p* = 0.0020) reached statistical significance. In the multivariate logistic regression analysis, only the MHR (OR: 1.0755 [95% CI: 1.0010–1.1556]; *p* = 0.0470) was found to be an independent marker for the prediction of PCOS.

**Table 3:** Possible confounding factors associated with monocyte counts to HDL-C ratio

Variables	Univariate logistic regression analysis		
	OR	95% CI	*P* value
Age (year)	1.0566	0.9818 to 1.1370	0.1368
Body mass index (kg/m^2^)	1.1600	1.0758 to 1.2509	< 0.0001
Systolic blood pressure (mmHg)	1.0209	0.9930 to 1.0496	0.1370
Diastolic blood pressure (mmHg)	1.0986	1.0525 to 1.1468	< 0.0001
HOMA-IR	1.9183	1.3913 to 2.6449	< 0.0001
Glucose (mg/dl)	1.0033	0.9738 to 1.0336	0.8296
hsCRP (mg/l)	1.2107	1.0181 to 1.4396	0.0211
Leucocytes (/mm^3^)	1.4282	1.1749 to 1.7361	0.0001
Neutrophil (/mm^3^)	1.6002	1.1971 to 2.1389	0.0004
Lymphocytes (/mm^3^)	1.8149	1.0584 to 3.1119	0.0238
Trigliserid (mg/dL)	1.0020	0.9965 to 1.0074	0.4713
Total cholesterol (mg/dL)	1.0093	0.9968 to 1.0220	0.1406
LDL (mg/dL)	1.0192	1.0053 to 1.0333	0.0041
Total testosteron (ng/dl)	1.9936	1.2264 to 3.2408	0.0024
FSH (IU/L)	1.0346	0.8252 to 1.2972	0.7681
LH (IU/L)	1.3157	1.1552 to 1.4985	< 0.0001
Estradiol (pg/ml)	1.0203	0.9934 to 1.0480	0.1297
TSH (mIU/L)	0.9632	0.6983 to 1.3286	0.8192
Prolaktin (ng/ml)	0.9889	0.9407 to 1.0395	0.6599

**Table 4:** Univariate and multivariate logistic regression analysis showing the predictors for the presence of polycystic ovary syndrome

Variables	Univariate logistic regression analysis			Multivariate logistic regression analysis		
	OR	95% CI	*P* value	OR	95% CI	*P* value
Age (year)	0.9863	0.9199 to 1.0575	0.6977	–	–	–
Body mass index (kg/m^2^)	1.0594	0.9934 to 1.1298	0.0744	0.9838	0.9077 to 1.0663	0.6904
HOMA-IR	1.3133	1.0543 to 1.6358	0.0067	1.1212	0.8660 to 1.4515	0.3854
hsCRP (mg/l)	1.2996	1.0804 to 1.5634	0.0023	1.2271	0.9970 to 1.5102	0.0534
Leucocytes (/mm^3^)	1.1091	0.9481 to 1.2973	0.1894	–	–	–
MHR	1.1006	1.0314 to 1.1745	0.0020	1.0755	1.0010 to 1.1556	0.0470

## Discussion

In the present study, we evaluated multiple clinical and biochemical variables in PCOS patients and compared them with those of age- and BMI-matched non-PCOS subjects. Our clinical and biochemical parameters confirmed that women with PCOS, had higher scores in terms of WHR, hirsutism, diastolic blood pressure, insulin, HOMA-IR, hsCRP, total cholesterol, LDL-C, total testosterone, and LH but also lower HDL-C values. Additionally, a subgroup analysis revealed that obese PCOS patients had higher levels of diastolic blood pressure, insulin, HOMA-IR, hsCRP, and monocytes, while members of the lean PCOS group had higher levels of diastolic blood pressure, insulin, HOMA-IR, hsCRP, neutrophils, lymphocytes, monocytes, and total testosterone as compared to age- and BMI-matched controls; notably, these findings were consistent with those of many previous studies [3, 20–24]. Importantly, we found that the MHR was significantly higher in women with PCOS than in women in the control group. To the best of our knowledge, this is the first study to investigate the association between the MHR and PCOS.

PCOS is a complex endocrine and metabolic disease that is associated with obesity, insulin resistance, compensatory hyperinsulinemia, and chronic low-grade inflammation [3]. Previous studies have shown that various biomarker alterations are associated with insulin resistance and low-grade inflammation in patients with PCOS [21, 22]. Therefore, PCOS is linked to a combination of risk factors that may lead to the development of cardiovascular disease and type 2 diabetes mellitus. Indeed, low-grade inflammation, impaired glucose tolerance, central obesity, dyslipidemia, and hypertension in PCOS patients are associated with an increase in cardiovascular disease risk [20].

The activation of monocytes and their differentiated forms into lipid-laden macrophages plays a significant role in the promotion of immune defenses in patients with chronic inflammatory conditions; these actions also drive inflammation and cardiovascular disease. Johnsen et al. demonstrated that an elevated monocyte count can be used as an independent predictor of future plaque development in previously plaque-free arteries [25]. Moreover, the activation of monocytes and their differentiated forms into macrophages can be modulated by inflammatory cytokines [26]. However, HDL-C molecules have the ability to counteract macrophage migration and eliminate cholesterol from these cells. Classically known as an antiatherogenic lipoprotein, HDL-C promotes reverse cholesterol transport from the arterial wall-specifically from lipid-laden macrophages [27]. It has been suggested that HDL-C may play a protective role in atherogenesis via the regulation of endothelial adhesion molecule expression, thereby preventing the generation of oxidatively modified LDL-C and the stimulation of endothelial nitric oxide synthases [27]. Additionally, HDL-C has anti-inflammatory, anti-oxidant, and anti-thrombotic effects [10, 27]. The anti-inflammatory effect of HDL-C results from its interaction with both circulating cells, which have the ability to inhibit both leukocyte and platelet activations. HDL-C is highly effective in inhibiting the endothelial expression of adhesion molecules and in preventing monocyte recruitment to the artery wall [28]. As briefly mentioned above, higher monocyte counts and lower HDL-C levels seem to be indirect indicators of inflammation and of the development of atherosclerosis [26]. Indeed, the relationship between these two parameters provides a better understanding of concomitant inflammation.

Relevant literature contains a limited number of studies on the MHR and its role in predicting inflammation. A recent study conducted by Cetin et al. found that the MHR was a novel inflammation-based marker that could be used as an independent predictor of both the severity of coronary artery disease and the likelihood of future cardiovascular events in patients with acute coronary syndrome [14]. Similarly, increases in the MHR have been associated with more severe cardiovascular prognoses in patients with chronic kidney disease [12]. Additionally, the MHR was an independent variable for cardiovascular events within this population [28]. Cockerill et al. indicated that the MHR may be a predictor of stent thrombosis and mortality in ST elevated myocardial infarction (STEMI) patients [29]. Moreover, a recent study conducted by Vahit et al. demonstrated that an increased MHR score may be a useful indicator of metabolic syndromes [18]. Consistent with these studies, we found that the average MHR value of patients with PCOS was substantially higher than that of patients without PCOS. Moreover, the univariate regression analysis showed that BMI, diastolic blood pressure, HOMA-IR, and CRP, were possible confounding factors affecting MHR values in our study population. Also, in an effort to identify independent variables for the prediction of PCOS, univariate and multivariate logistic regression analyses were conducted; these analyses showed that the MHR was the only independent variable for women with PCOS. These results indicated that higher MHR values may be associated with ongoing inflammation in the pathophysiology of PCOS. In their entirely, these findings signaled the importance of the MHR with regard to systemic inflammatory conditions and endothelial dysfunction, both of which play crucial roles in the development of future cardiovascular disease.

Insulin resistance is a cornerstone of PCOS. Insulin resistance and compensatory hyperinsulinemia in PCOS patients seem to have a stimulatory effect on chronic low-grade inflammation. It is known that obesity is associated with insulin resistance and low-grade inflammation in the PCOS population [24]. Fat tissues, such as those found in endocrine organs, engage in metabolic and endocrinological activities whereby cytokines and other messenger proteins called adipokines are produced and secreted. Cytokines mediate many physiological and pathological metabolic processes, such as satiety and energy balance, inflammation, insulin resistance/sensitivity, angiogenesis, lipid metabolism, and atherosclerosis. Many previous studies have shown that early atherogenesis and subclinical atherosclerosis are more common in patients with PCOS than in control subjects [30]. In line with the existing literature, we found that obese PCOS patients had higher HOMA-IR, hsCRP, and total leucocyte counts but lower HDL-C levels than did lean PCOS patients [3, 20–24]. Moreover, the MHR values of obese PCOS patients were significantly higher than those of lean PCOS patients. Additionally, the HOMA-IR and total leucocyte counts were significantly higher in obese control patients than lean control patients. As expected, the MHR values in obese control patients were higher than in lean control patients in our study population. These results clearly indicated that obese PCOS patients tend to be at a higher risk for the development of future cardiovascular disease than are other patients.

### Limitations

The main limitations of this study concerns its cross-sectional study design and its relatively small population size. Moreover, monocyte and lymphocyte counts were calculated automatically using peripheric blood samples. The absence of other inflammation and oxidation parameters, in conjunction with the lack of a comparison to other inflammation biomarkers, was another limitation of this study. Data regarding patients’ alcohol intake and smoking habits to include frequency of use and years of smoking history, both of which may affect the development of PCOS, were not included in our study due to the absence of records.

## Conclusions

This study was the first to investigate the significance of the MHR in patients with PCOS. Our study demonstrated that patients with PCOS have higher MHR values than do women without the condition. The MHR may be used as a simple, low-cost, reproducible biochemical marker to be used in the detection of cardiovascular disease risk in PCOS patients; notably, it is also beneficial in the diagnosis of PCOS using other diagnostic criteria. The present study suggests that further prospective randomized studies be conducted in order to fully evaluate the association between the MHR and PCOS.

## Abbreviations

BMI: Body mass index
CVD: Cardiovascular disease
DM: Diabetes mellitus
E2: Estradiol
ELISA: Enzyme-linked immunosorbent assay
FSH: Follicle-stimulating hormone
HDL: High-density lipoprotein
HOMA-IR: Homeostatic model assessment for insulin resistance
hsCRP: High-sensitivity C-reactive protein
LDL: Low-density lipoprotein cholesterol
LH: Luteinizing hormone
MHR: Monocyte counts to high-density lipoprotein cholesterol ratio
OR: Odds ratio
PCOS: Polycystic ovary syndrome
SD: Standard deviation
STEMI: ST-segment elevation myocardial infarction
TSH: Thyroid-stimulating hormone
